# Design and measurement of 3D-printed variable-morphology and variable-density breast phantoms for mammography and breast CT dose assessment

**DOI:** 10.1186/s41205-026-00335-9

**Published:** 2026-07-11

**Authors:** Catherine Paverd, Gianluca Piol, Davide Cester

**Affiliations:** 1 Institute for Diagnostic and Interventional Radiology, University Hospital Zurich, Rämistrasse 100, Zurich, 8091 Switzerland; 2 University of Zurich, Rämistrasse 71, Zurich, 8006 Switzerland

**Keywords:** 3D-Printed phantoms, Breast computed tomography, Mammography, Monte carlo simulation, Radiation protection

## Abstract

Accurate dose quantification across radiation-based breast imaging techniques is important for patient safety, but currently available phantoms do not easily allow the assessment of the impact of breast size and density across modalities. Here we design, produce, and validate three dimensional (3D)-printed, variable-morphology, fillable phantoms for the purpose of radiation dose measurements and comparison studies. Three representative breast shapes (small, medium, large) were exported from real Breast Computed Tomography (BCT) data. Corresponding compressed shapes were generated following the Virtual Imaging Clinical Trials for Regulatory Evaluation (VICTRE) pipeline, in which morphology is controlled through 10 separate length and deformation parameters, optimized to match uncompressed phantom volumes. Surface models of each shape were printed using a 3D printer (Stratasys F370-3D, alphacam GmbH). Four mixtures, representative of Breast Imaging-Reporting and Data System (BI-RADS) categories A-D, were prepared using varying ratios of 1.5% agarose gel (agar), rapeseed oil, and soy lecithin, poured into the printed surface models, and set overnight at $4^{\circ}$C. Pre-calibrated dosimeters were placed on the irradiated surface of each phantom to measure Entrance Surface Dose (ESD). Compressed phantoms in all sizes and densities were successfully measured in a mammography device (Senographe Essential, GE Healthcare, DE). Uncompressed phantoms in all sizes and in three of the four densities were successfully measured in a BCT device (nu:view, Advanced Breast-CT). Measured ESD matched the values obtained with Monte Carlo simulations; for the unit equipped with automatic exposure control, the ESD increased with increasing breast size and with increasing breast density, confirming the known correlation between thickness, density, and ESD and validating the capabilities of the proposed phantoms to represent breasts with real physical properties.

**Supplementary Information:**

The online version contains supplementary material available at 10.1186/s41205-026-00335-9.

## Introduction

Breast cancer is the leading cause of cancer-related deaths in women globally [1]. Screening programs enable the detection of breast cancer at an early stage, thereby reducing breast cancer-related mortality [2]. The standard breast cancer screening technique is two-view digital mammography for each breast, which is a well-established, low-dose imaging technique, but which has limitations in terms of lack of sensitivity in dense breasts as well as patient discomfort.

Recently, new protocols, such as Digital Breast Tomosynthesis (DBT) protocols, and breast-specific devices, such as Breast Computed Tomography (BCT) devices, have been introduced to address some of the limitations of mammography screening. These techniques improve image quality or reduce patient discomfort in breast cancer screening; however, in some cases they may increase radiation dose compared to conventional 2D mammography scans [3–5]. For example, a national retrospective study in Switzerland demonstrated that DBT was associated with higher Mean Glandular Dose (MGD) in the breast compared to conventional (2D) mammography [6], and in a review of the dose in Sweden’s national screening program, Skaane et al. reported that the MGD in DBT procedures was approximately 23% higher than in conventional mammography scans [7]. The difference in dose levels between conventional mammography and DBT scans is also manufacturer-dependent, with MGD for DBT ranging from 1.1 up to 1.9 times higher than in conventional mammography, depending on manufacturer protocols [8]. For BCT, MGD is also increased relative to conventional mammography, with Vedantham et al. reporting a 13.5% increase in the median MGD [9]. However, with newer photon-counting detectors, it is possible that significant dose reduction could be achieved in BCT devices, as shown in a study on surgical specimens [10] and in simulation [11]. Given that radiation dose deposition in the breast should be minimised wherever possible, a method for accurate dose quantification across radiation-based breast imaging devices is important to advance understanding of dose deposition and minimise radiation-induced cancer risk in patients with different breast sizes and densities.

One method of assessing radiation dose in a controlled manner is through phantom studies. However, currently available phantoms do not easily allow the assessment of the impact of breast size and density across modalities.

In the case of mammography, most phantoms are designed for quality assurance purposes, including, for example, contrast detail phantoms such as the Contrast Detail for Mammography (CDMAM) phantom [12], or phantoms containing objects that simulate microcalcifications and masses, such as the North American College of Radiology (ACR) phantom [13]. The CDMAM phantom contains 672 gold discs divided into 21 exponential steps, with diameters ranging from 0.08 to 2.0 mm, and has various thicknesses of polymethyl methacrylate (PMMA) covers [12]. The ACR phantom includes objects from 0.16 to 2.0 mm, and also includes several PMMA plates to enable different thickness assessments, as well as an acrylic disk to establish density differences [13]. Another phantom, produced by the Computerized Imaging Reference Systems group (CIRS, owned by Sun Nuclear, FL, USA) is the BR3D Breast Imaging Phantom, which is designed to assess detectability of lesions of various sizes within a tissue equivalent, heterogeneous background, which is generated by “swirling” together tissue-equivalent materials of 100% adipose and 100% glandular tissue density in an approximate 50/50 ratio by weight [14]. This phantom also has fixed morphology optimised for mammography, but the internal pattern is unique to each phantom, limiting its use as a standardised phantom. In all of the reference phantoms listed, the thickness of the phantoms can be adjusted, but the morphology and density are constant.

In the case of BCT, dose measurements have primarily been examined in pure simulation studies [11, 15], in simulation studies with comparison to excised breast tissue [16], or in phantom studies using slab or half-ellipsoid shaped PMMA phantoms [17, 18]. Custom phantoms have also been designed for dose assessment, for example, Hernandez and Boone manufactured six phantoms of different volumes using data derived from real BCT patient data [19]. The custom phantoms were manufactured using Ultra-high-molecular-weight polyethylene (UHMW) as it has a density similar to that of adipose tissue in the breast (0.930 g/cm^3^), however the density of the phantoms was not adjustable after manufacture [19].

Finally, other custom breast phantoms with more complicated geometries have been manufactured using 3D printing, however they are not optimised for radiation dose quantification, but rather for anatomical visualisation [20, 21]. In particular, commonly used printing materials (such as Acrylonitrile Butadiene Styrene (ABS), Polyethylene Terephthalate (PET), Polyvinyl Alcohol (PVA), and photopolymer resins) do not exhibit fully accurate tissue equivalence in terms of energy-dependent X-ray attenuation, leading to discrepancies in radiation absorption relative to adipose and glandular breast tissues [22, 23]. However, Belarra et al. showed that in some cases, 3D-printing materials could show similar absorption characteristics overall to breast tissue with different ratios of glandular tissue [24].

Several approaches exist for 3D printing anthropomorphic phantoms. For example, Carton et al. used a two compartment approach by 3D-printing the glandular tissue, skin, and Cooper’s ligaments using a material with 50% glandular radiographic equivalence (FC-720), and then permanently filling the remaining adipose compartments with a 100% adipose equivalent epoxy-based resin [25]. Other groups used a similar approach, combining 3D-printed PET structures with various filling materials (beeswax, resin, and oil) for mammography [26], or with water for BCT [27], but in some cases the resulting images showed artefacts due to internally trapped air [26]. More complicated printing approaches aim to combine multiple 3D-printing materials in a single phantom. For example, Kiarashi et al. printed phantoms with realistic breast imaging qualities using two materials (TangoGray and TangoPlus) [26], and di Franco et al. and Varallo et al. showed qualitatively good imaging results for BCT phantoms printed with Nylon, ABS and PVA [28], and mammography phantoms printed with ABS and PET [28, 29]. However, these anthropomorphic phantoms were not evaluated for radiation dose, rather for qualitative image quality.

Thus existing phantoms cannot accurately recreate variable breast morphology (size and shape) and variable breast density with the purpose of measuring radiation dose across different breast cancer screening devices. To address this, we design, produce, and validate low-cost, 3D-printed, variable-morphology, fillable phantoms for the purpose of radiation dose measurement and comparison studies.

## Materials and methods

### Phantoms

Data from 48 patients who received both mammography and Breast CT exams between January 2021 and June 2022 was fully anonymised and exported for further processing. Full anonymisation ensured that there was no way to re-identify individual patients from their scans. According to the Swiss Federal Act on Research involving Human Beings (Human Research Act, HRA), research conducted on irreversibly anonymised data does not require ethical approval and thus, in accordance with institutional guidelines, no ethics committee approval or informed consent was required for this study. The scans represented a variety of breast shapes, sizes, and densities, as well as varying mammography compression thickness. Phantom shapes based on three real scans were selected as representative shapes for small, medium, and large breasts; these shapes served as the BCT uncompressed models for 3D printing, while their volume and compression thickness were used to generate the corresponding mammography compressed models.

#### Uncompressed phantom shape generation

The three selected BCT scans were imported into 3D Slicer [30] and converted into empty surfaces, imposing an edge thickness of 2 mm. Thereafter, a flat border was added on the open edges of the uncompressed phantoms to facilitate their handling. Finally, small structures that were not needed in the experiment, such as ducts extending into the chest region, were manually removed, taking care to avoid the introduction of holes of any kind on the surface during each stage.

#### Compressed phantom shape generation

Compressed shapes were generated using the Virtual Imaging Clinical Trials for Regulatory Evaluation (VICTRE) Pipeline (developed by the Division of Imaging, Diagnostics, and Software Reliability at the U.S. Food and Drug Administration) [31]. The VICTRE software contains modules for breast phantom generation, compression, and cropping, which were used to generate the compressed phantoms. To generate a breast in VICTRE, transformations are applied to a base superquadratic surface. The breast shape model has previously been described in detail [32, 33]. In summary, size parameters adjust the breast volume in the top, bottom, left hemisphere, right hemisphere, and length dimensions. Deformation parameters control the quadric deformation along the polar angle, the ptosis deformation (which allows for age-induced sagging of a breast), and the turn-pop deformation (which allows the top part of the virtual breast to stretch in the direction of the shoulder). Other important parameters are the voxel size (which controls the balance between spatial resolution and computing power) and the desired percentage of fat tissue. The output of the generation process is a voxelized volume where each voxel is associated with a specific tissue (air, fat, skin, glandular, nipple, muscle, ligament, Terminal Duct Lobular Unit (TDLU), duct, artery, or vein).

To generate phantoms that were directly comparable with the real scans, the shape parameters of the virtual phantoms were optimized such that the initial, free-hanging, uncompressed shapes had the same volumes as the representative uncompressed breasts derived from real scans. To achieve this, the ratios between all the size parameters of the breast shape model were kept constant, and the parameters were linearly increased from the default values in steps of 5% until the volume reached the target volume.

These volume-matched uncompressed virtual phantoms were then used as input to the breast compression module in VICTRE, which uses an external finite element solid mechanics software (Finite Elements in Biomechanics - FEBio [34]) to model breast compression between two planar surfaces. Voxelized breast phantom files were loaded into the module and a tetrahedral mesh was generated for each breast, with linear elasticity properties assigned to each tetrahedron based on the corresponding original material. These properties were optimized as part of the VICTRE project and have been used unaltered; they are reported in Table 1.

**Table 1:** Elastic properties of virtual breast materials, as determined by the VICTRE project

		[kg/mm${}^3$]	[KPa]	
Material	Type	Density	Young’s modulus	Poisson’s ratio
Fatty tissue	neo-Hookean	1e-06	5	0.49
Glandular tissue	neo-Hookean	1e-06	15	0.49
Muscle	neo-Hookean	1e-06	5	0.49
Breast masses	neo-Hookean	1e-05	20	0.49
Paddle material	solid body	1e-05	-	-

As the next step in the compression process, virtual compression plates were then generated above and below the breast phantom, and moved towards each other until the desired thickness was achieved. The desired thickness value was determined through comparison of thickness versus compression force from real patient data. Specifically, the compression force data from 38 patients who had undergone both BCT and mammography examinations was analysed; breast volumes were calculated from the CT dataset, while the mammography data provided force and thickness values. 10 examinations with force values in excess of $\pm$25% from the reference value of 100 N were excluded, and a linear regression fit was used to determine the relationship between breast volume and resulting thickness.

The output of the compression module in VICTRE was a voxelized phantom saved in the same file format as the generated breasts. The voxelised phantom was then loaded into 3D Slicer in order to add a flat surface on the posterior (chest wall) side, and an upper opening to allow for filling.

#### Phantom printing and impermeabilization

The surface models of the six phantoms were printed using a Fused Deposition Modelling (FDM) 3D printer (Stratasys F370, alphacam swiss GmbH) with high-strength ABS Ivory filament. ABS was selected for this study not only for its physical density (which is approximately in the range of the density of human skin), but also for its X-ray attenuation properties. Unlike materials chosen solely based on mass density, ABS exhibits an effective atomic number that closely mimics human soft tissue within the diagnostic energy range. This selection aligns with established works in literature, which identify ABS as a reliable tissue-equivalent material for the fabrication of anthropomorphic phantoms, ensuring superior dosimetric accuracy compared to other common polymers [35–38]. Following printing, the resulting 3D-printed phantoms were coated with a thin layer of water-resistant wood glue to make them watertight. The effect of the painted glue on overall radiotransparency was negligible, as determined experimentally by comparison of the Hounsfield units for CT scans between a coated and an uncoated slab.

#### Phantom filling

According to the 4th edition of the American College of Radiology (ACR) BI-RADS classification, breast densities can be assigned to one of four categories, from A to D; although the classification system has subsequently changed in the 5th edition of the ACR BI-RADS, in this work a filling mixture representative of each of the four A - D categories was prepared in a similar manner to that described in Germann et al. [39]. Each of the mixtures contained the following components in different proportions: 1.5% agar solution (Bacto Agar, Becton Dickinson), rapeseed oil (COOP Switzerland GmbH), and soy lecithin (Soleil Vie - Montasell SA). The agar solution represented fibroglandular breast tissue, whereas the rapeseed oil represented fatty tissue. A summary of the BI-RADS 4th edition classification and the corresponding quantities of agar, oil and lecithin are reported in Table 2.

**Table 2:** Variable density filling mixture properties

Mixture	1	2	3	4
BI-RADS description	fatty	sparsely	dense	very
		dense		dense
BI-RADS category	A	B	C	D
BI-RADS fibrograndular tissue	<25%	25–50%	50–75%	>75%
Filling non-fat content (glandularity)	12.5%	37.5%	62.5%	87.5%
− 1.5% Agar solution	119 ml	356 ml	594 ml	831 ml
- Rapeseed oil	831 ml	594 ml	356 ml	119 ml
- Soy lecithin	50 ml	50 ml	50 ml	50 ml
Filling volume (normalized)	1 l	1 l	1 l	1 l
Filling consistency	liquid	gel/liquid	gel	gel

**Fig. 1:**
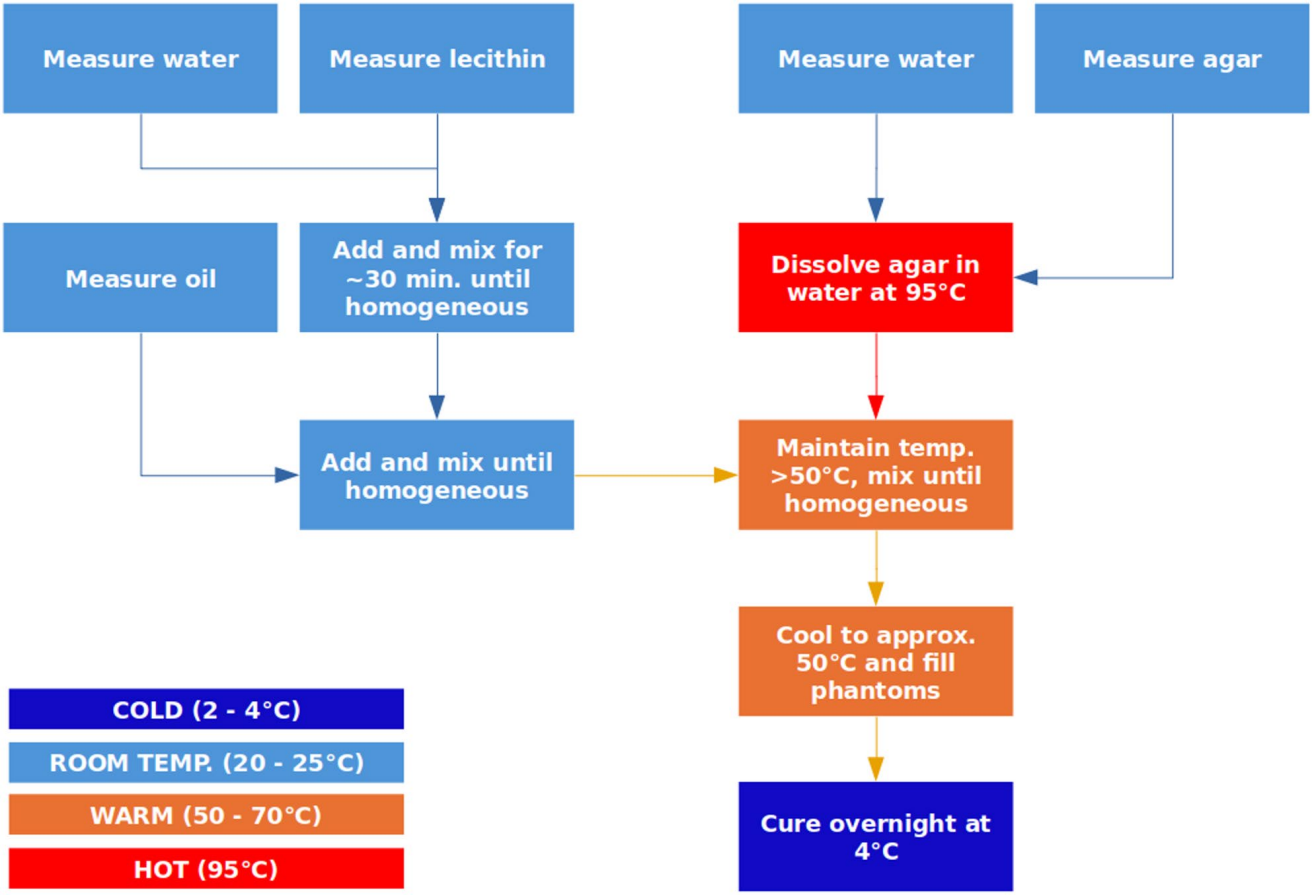
Block diagram of the phantom filling preparation process

Figure 1 shows a block diagram of the mixture preparation process. First, the lecithin solution was prepared according to the instructions on the package, mixed with the oil, and then stirred until the liquid became homogeneous. Separately, a 1.5% by weight agar solution was obtained by mixing agar powder with water heated to $95^\circ$ C. The agar solution was left to cool with continual stirring until it turned opaque, at which point the oil and lecithin mixture was slowly added. When the resulting mixture reached a temperature of around $50^\circ$ C, it was poured into the 3D-printed breast surfaces, left to cool to room temperature, and then stored at $4^\circ$ C overnight. Following curing, the BI-RADS category C and D mixtures solidified, allowing for easy transportation and manipulation of the phantoms. In the category B mixture, some oil did not fully bind with the agar and remained liquid, although the phantoms could still be manipulated with some precaution against spilling. The BI-RADS category A mixture consisted of sparse agar aggregates suspended in a fully liquid oil bath.

### Dosimetry

Phantom measurements were performed using a MOSFET (Metal Oxide Semiconductor Field Effect Transistor) dosimetry system (TN-1002RD, Best Medical Canada, Ontario, CA), which allows for the monitoring of five MOSFET dosimeters in real time [40]. The dosimeters had a dose range of 7000 cGy, sensitivity of 30 mV/R, active region of 0.2 $\times$ 0.2 mm, and dose-to-dose reproducibility of 3%. The system was designed to be used in both therapy and diagnostic applications, and its suitability for low-dose, low-keV measurements has been independently validated [41].

Dosimeters were individually calibrated prior to measurement by placing the black surface facing the radiation source and not using any build-up material (as recommended in the manual). BCT calibrations were performed on a CT scanner (NAEOTOM Alpha, Siemens Healthineers) to allow greater flexibility in controlling scan parameters. Dosimeters were exposed to different doses in the range of 5 – 40 mGy, using a CT ionization chamber as reference (DCT10, IBA Dosimetry [42]). Calibrations were performed with Automatic Exposure Control (AEC) deactivated, and the maximum energy was set to the minimum value available (120 kV). Mammography calibrations were performed on the same device using a dedicated mammography dosimeter as reference (Multi Detector XM, IBA Dosimetry); the dosimeters were exposed to multiple mammography irradiations with dose values in the range 5 – 20 mGy. A quality assurance phantom (MTM100, Meditest, France) was used to provide the necessary attenuation for the AEC system to work in realistic conditions.

### Measurement protocol

For each desired breast density, two litres of the appropriate mixtures were prepared the day before each measurement. This quantity was sufficient to fill all the phantoms at the same time. Between measurements, the filling mixtures were extracted and stored in a refrigerated environment, and the phantoms were cleaned with water. Measurements were performed on a mammography device (Senographe Essential, GE Healthcare, DE) and a BCT device (nu:view, Advanced Breast-CT, Erlangen, DE), with characteristics given in Table 3.

**Table 3:** Main characteristics of the two breast imaging systems compared in this work. Al = Aluminium, Be = Beryllium, Mo = Molybdenum, Re = Rhenium, Rh = Rhodium, W = Tungsten; eq. = equivalent

	Mammography (MG) [43]	Breast-CT (BCT) [44]
Manufacturer	GE Healthcare	Advanced Breast-CT
Model	Senographe Essential	nu:view
Entry into service	2015	2018
Detector technology	Semiconductor	Direct conversion
Detector type	Amorphous Silicon	Cadmium Telluride
Detector area	240$\times$310 mm${}^2$	280$\times$50 mm${}^2$
Pixel size	(100 μm)${}^2$	(100 μm)${}^2$
Tube voltage	22 - 49 kVp	60 kVp
Voltage selection	automatic	fixed
Target material	Rh	Mo with Re-W facing
Focal spot size	0.30 mm	0.45 mm
Filtration	Be (8 μm Al eq.), Rh (25 μm)	Be (3 mm Al eq.)
Tube current	2 - 100 mA	5 - 125 mA
Current modulation	automatic	manual (fixed current)

During mammography measurements, wood elements with heights matching the phantom thickness were placed at the edge of the compression pad on the gantry side, to allow the mammography system to apply the expected compression force to the wooden elements whilst preventing damage to the phantoms. Five dosimeters were placed next to each other on the top surface of the breast phantoms, approximately halfway along the chest-nipple axis (image of placement available in Supplementary Material). The dosimeters were spaced to cover an area of approximately 2 $\times$ 2 cm${}^2$, and enabled the direct measurement of the average Entrance Surface Dose (ESD).

Each phantom was scanned six times with a tomosynthesis protocol (TOMO-L-CC). The six measurements across all five dosimeters were averaged, and their standard deviation was taken as an experimental estimation of the measurement error. The mammography device included an AEC system, which optimized exposure parameters as a function of breast density and thickness.

After each measurement with the mammography unit, the uncompressed phantoms (filled with the same mixtures) were measured. A custom-made suspension mechanism was used to hold the phantoms in the appropriate position during the measurement (image available in Supplementary Material). For measurements, four dosimeters were placed in each quadrant of the phantom, at the midpoint between the chest wall and the nipple. All BCT scans were performed with High Resolution reconstruction, and scan lengths were defined as 80 cm for small phantoms, 120 cm for medium phantoms, and 160 cm for large phantoms. The BCT device was not equipped with an AEC system; instead, the tube current was manually selected following the recommendation contained in [39] to optimize the SNR and dose, resulting in a setting of 25 mA for all small and medium phantoms, and 32 mA for all large phantoms. Two consecutive BCT scans were performed for each phantom in order to reduce the measurement error. BCT ESD was defined as the average reading of both measurements across all four dosimeters, and the standard deviation was taken as an estimation of measurement error.

### Monte Carlo simulations

All the measurements were matched by Monte Carlo simulations performed with a commercial software package (ImpactMC, AB-CT GmbH, Germany). For mammography simulations, the compressed mammography phantom outputs from VICTRE, in conjunction with manually set scan parameters, were used as input to the Monte Carlo simulation. For BCT Monte Carlo simulations, the DICOM dataset and images acquired from the BCT device were directly used as input to the Monte Carlo simulation software. In both cases, simulation parameters were adjusted to match the different irradiation parameters (geometry, tube current, X-ray spectrum) obtained from the device documentation (for mammography) or from the DICOM headers (for BCT). Although the virtual phantoms were generated with a complete internal mapping of the tissue, for each simulation, the phantom content was replaced by a single homogeneous material with density equal to the nominal average density of the corresponding measured mixture. The Monte Carlo software used in this work requires that the Air Kerma value (AK) in reference conditions (100 mAs) is provided as input, to characterize the tube output and normalize the dose calculations. These values were obtained independently from the main measurements: for the Breast CT the Air Kerma is a simple, factory-calibrated function of the tube current in mA, while for the mammography unit the Air Kerma for 100 mAs was measured with the calibration dosimeters for all possible tube potential (kVp) values.

An overview of the Monte Carlo processing pipeline for the two different modalities is reported in Table 4.

**Table 4:** Summary of the Monte Carlo pipeline for the two modalities

Parameter	Mammography	Breast CT
Input object	VICTRE digital phantoms	DICOM data of phantom scans
Irradiation pattern	According to device documentation	According to DICOM scan metadata
Dose normalization (AK / 100 mAs)	Direct measurements for each used kV value	Constant and determined by the manufacturer

## Results

**Fig. 2:**
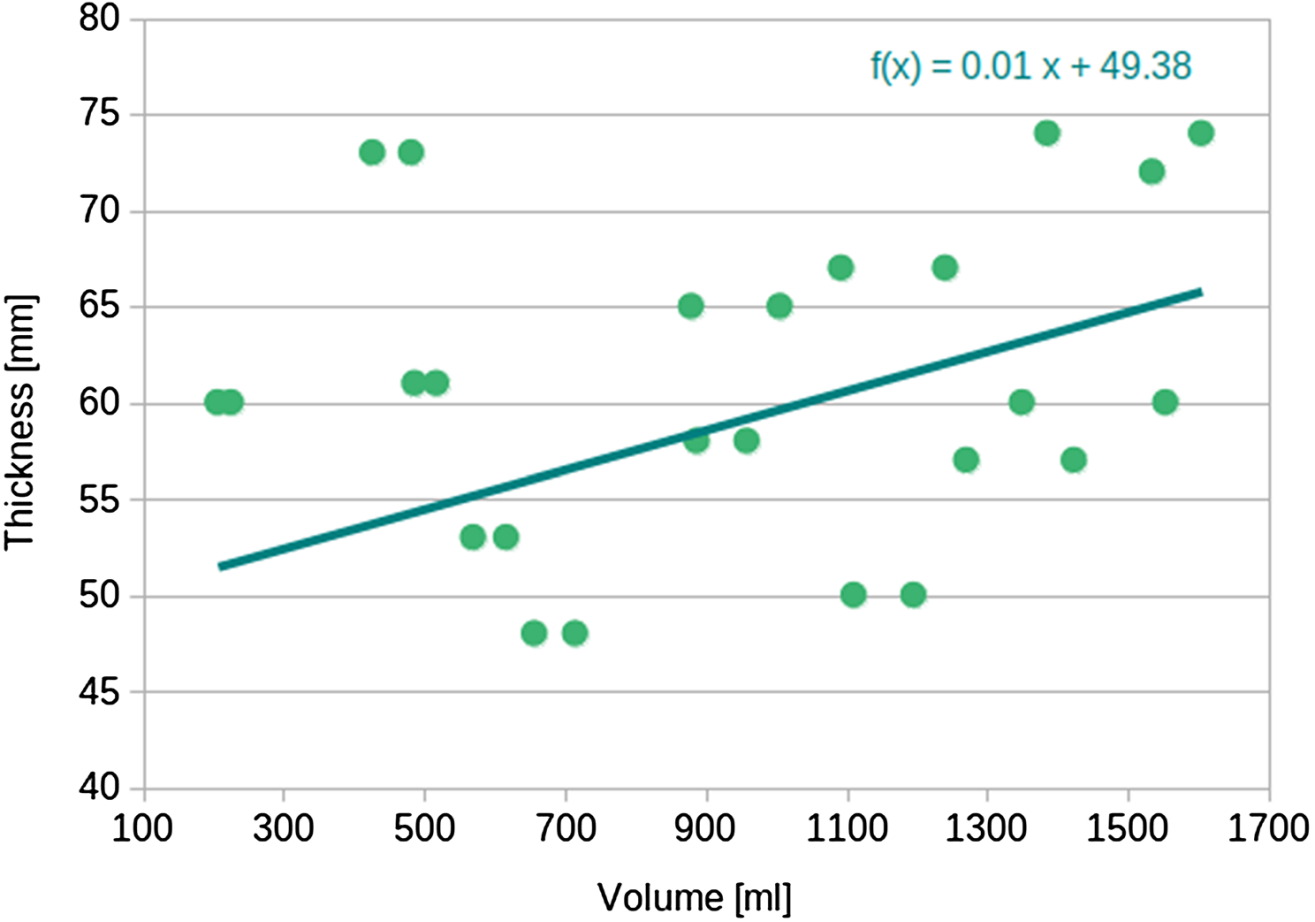
Compressed thickness as a function of the breast volume, for compression force in the range 100 N $\pm$ 25%. Data from 38 patient scans is shown, and volume measurements are given in millilitres (to match phantom filling volumes in millilitres)

Compressed phantom models in all three sizes and all four densities were successfully manufactured. Table 5 presents an overview of the features of the 3D-printed fillable phantoms presented in this work in comparison to the reference phantoms and phantoms presented in literature, in terms of external geometry, internal structure, density selection, utility for dosimetry measurements, and key limitations. Table 6 presents the summarized properties for the final printed phantoms, including the final scaling factor used during the virtual phantom generation process, and Fig. 2 shows the data used to determine the relationship between thickness as a function of breast volume.

**Table 5:** Summary of the main features of reference phantoms and phantoms reported in literature (rows 1 - 4) in comparison to the main features of the phantoms presented in this work (row 5)

Phantom type / approach	Geometric Realism	Internal Realism	Density selection	Dosimetry Use	Limitations
Simple slab / PMMA phantoms	Low	Low	None (fixed)	Yes	Not anatomically realistic, density fixed
Custom machined phantoms (e.g. UHMW)	Medium	Medium	None (fixed)	Yes	Density fixed
Hybrid phantoms (printed shell/internals plus filled compartments)	Medium - High	Improved (separate materials)	Medium - High	Limited	Not validated for dose; air artefacts
3D-printed anthropomorphic phantoms (single/multi-material)	High	High	Limited (discrete)	Limited	Complex fabrication; fixed internal structure
3D-printed fillable phantoms	High	Low	Continuous	Yes	Liquid handling, no internal structure

**Table 6:** Summary of the properties of the 3D-printed phantoms

	Small	Medium	Large
Real shapes, volume [ml]	246	641	1087
Virtual shapes, volume [ml]	247	640	1085
Virtual shapes, scaling factor	1.12	1.51	1.78
Virtual shapes, compression [mm]	53	57	61

**Fig. 3:**
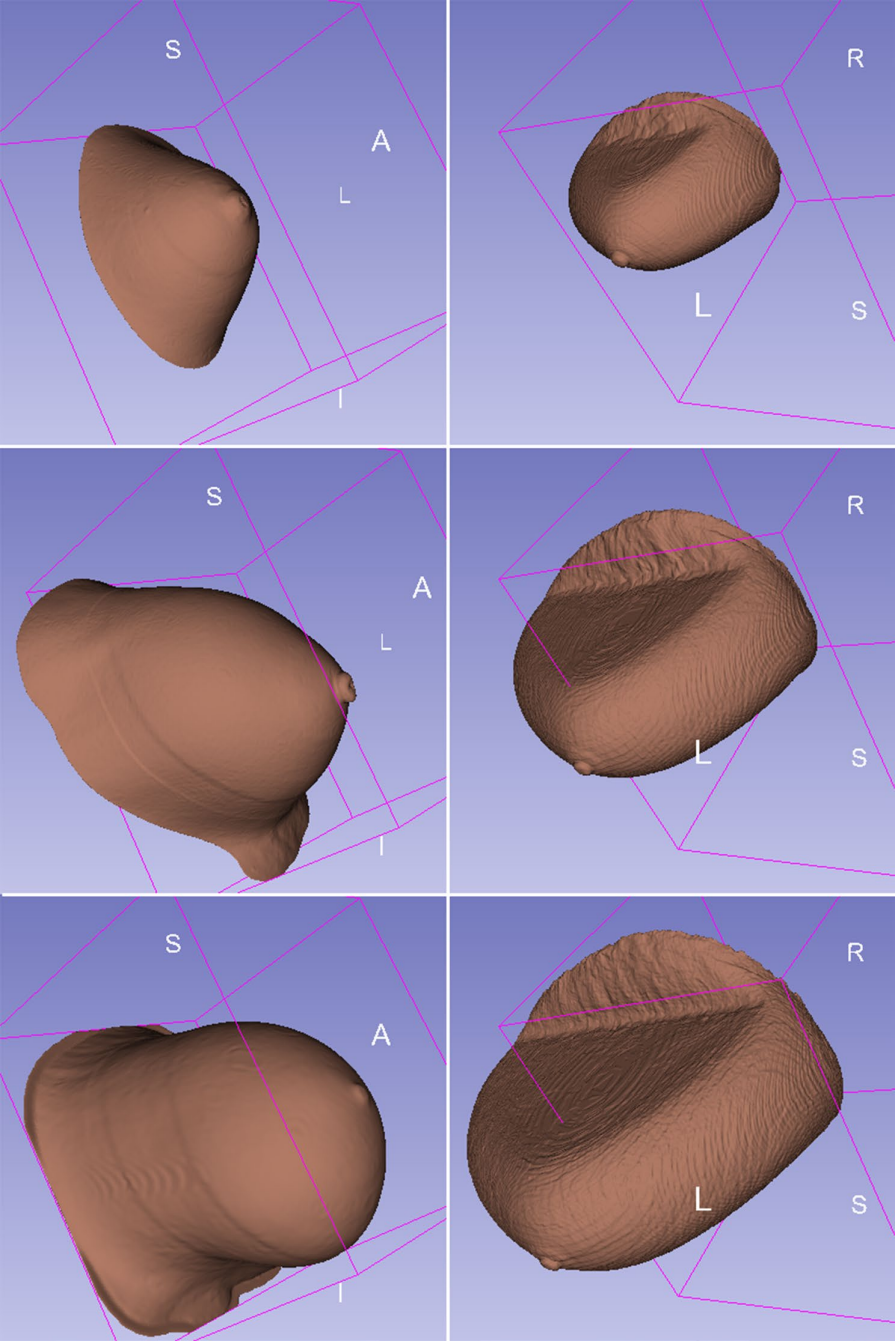
Oblique view of the 3D phantom surfaces shown in 3D Slicer: uncompressed (left) and compressed (right) shapes for small (top row), medium (middle row), and large (bottom row) breast sizes

**Fig. 4:**
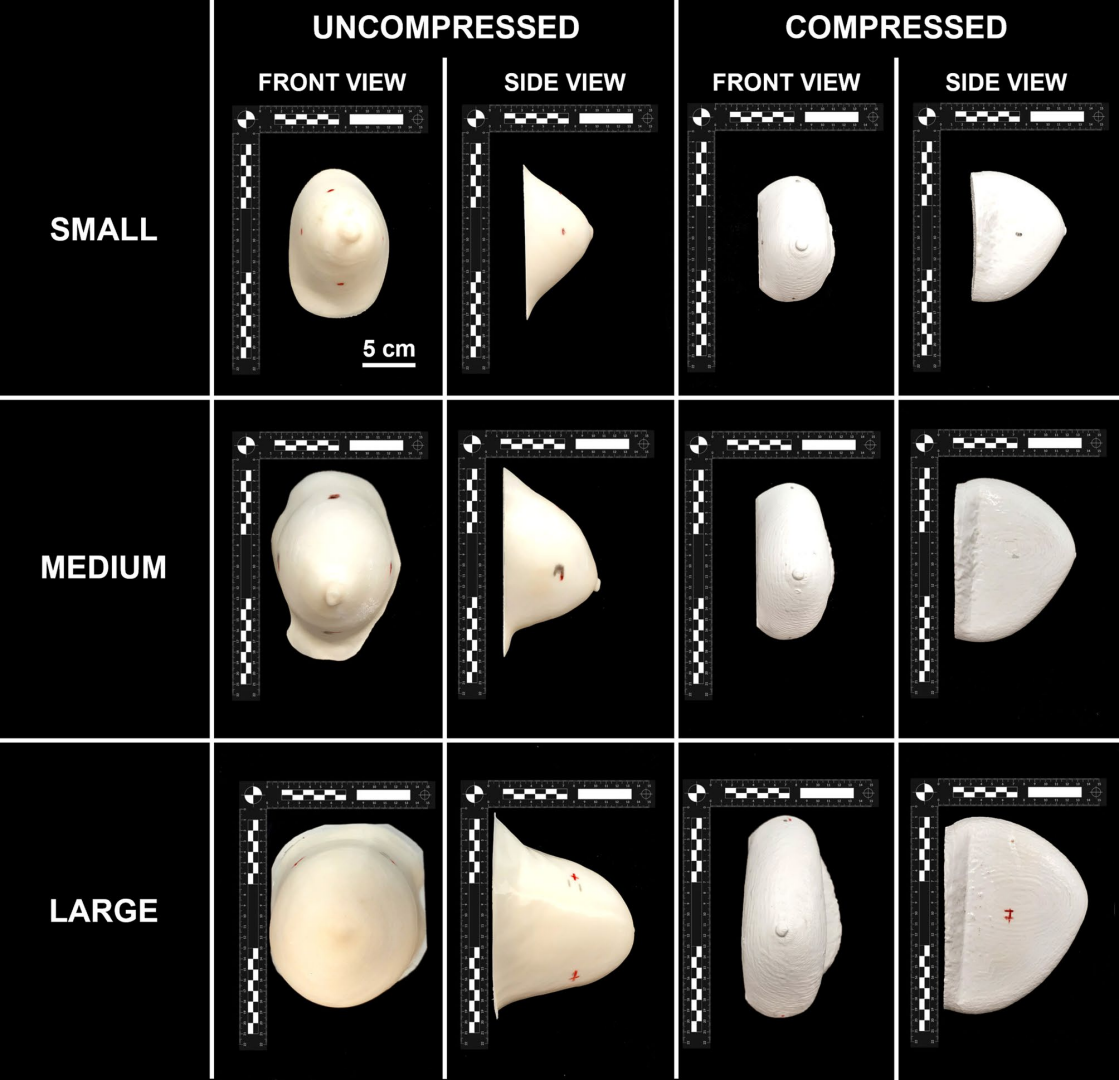
Photographs of the 3D-printed phantoms (not filled) from the front (columns 1 and 3) and lateral (columns 2 and 4) aspects. Images are of uncompressed (columns 1 and 2) and compressed (columns 3 and 4) phantoms for the small (top row), medium (middle row) and large (bottom row). Uncompressed and compressed phantoms have the same volume per size

Figure 3 provides oblique views of the 3D phantom surfaces during the simulation process for both free and compressed breast shapes for all sizes, visually demonstrating the amount of compression in the breast. Photographs with scale bars of the final printed models are presented in Fig. 4, showing the relative changes in size and shape for each phantom.

For measurements performed with the BCT device, uncompressed phantoms in all sizes and in three of the four filling densities were successfully measured. As shown in Table 2, the mixture corresponding to BI-RADS Category A (the lowest glandularity/highest fat content) remained in liquid form, precluding measurement in the BCT device due to risk of spillage and damage to the device. For measurements performed on the mammography device, compressed phantoms in all sizes and densities were successfully measured.

**Fig. 5:**
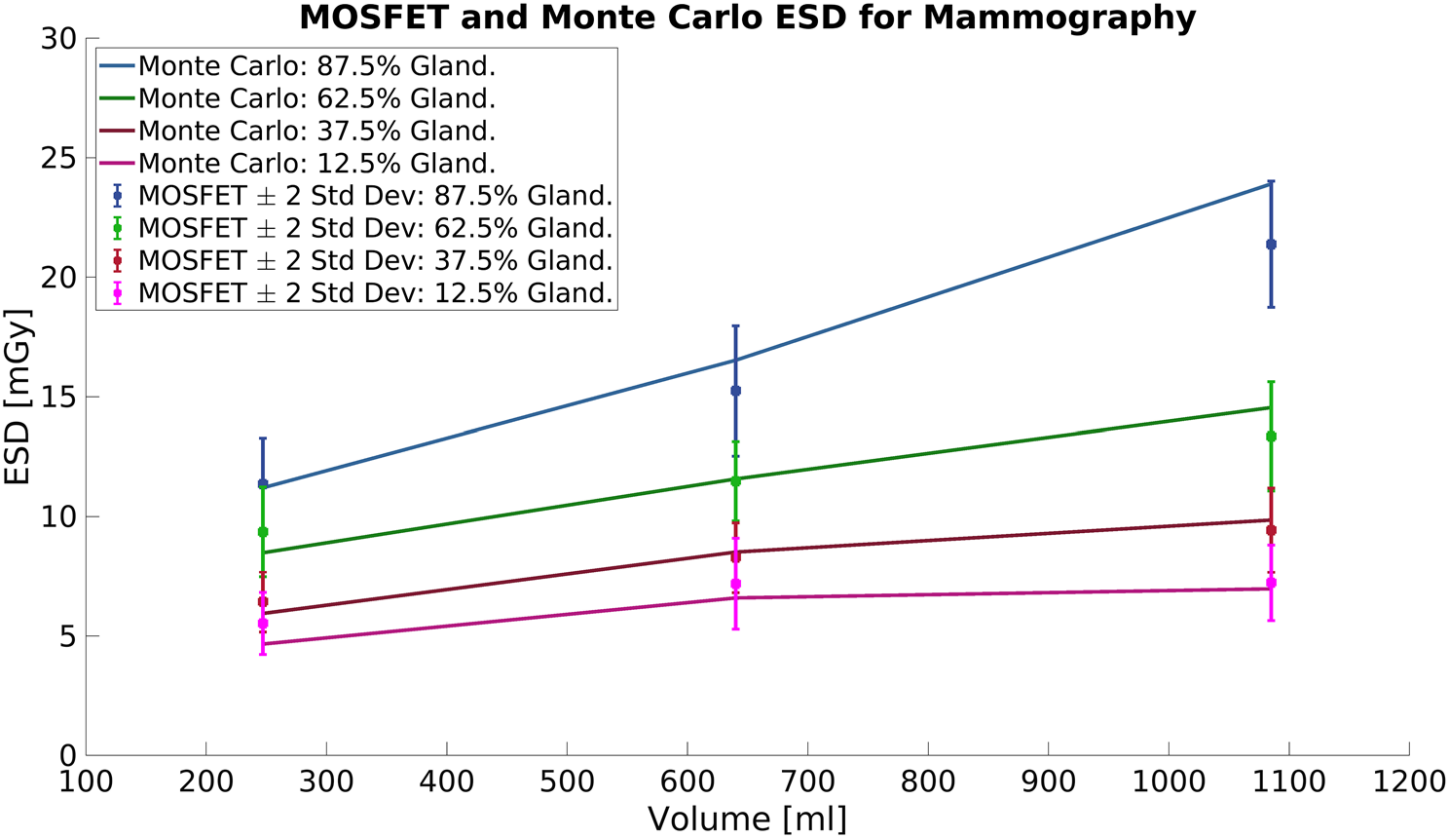
Entrance surface dose (ESD) for the mammography measurements for both simulated (solid lines) and measured (square markers) data, for all three phantom sizes and all four densities

Figure 5 presents the ESD values for the mammography device, both measured and simulated, for all four filling mixtures. The agreement between measurements and simulations is very good, with simulated values falling within two standard deviations of the measured values.

**Fig. 6:**
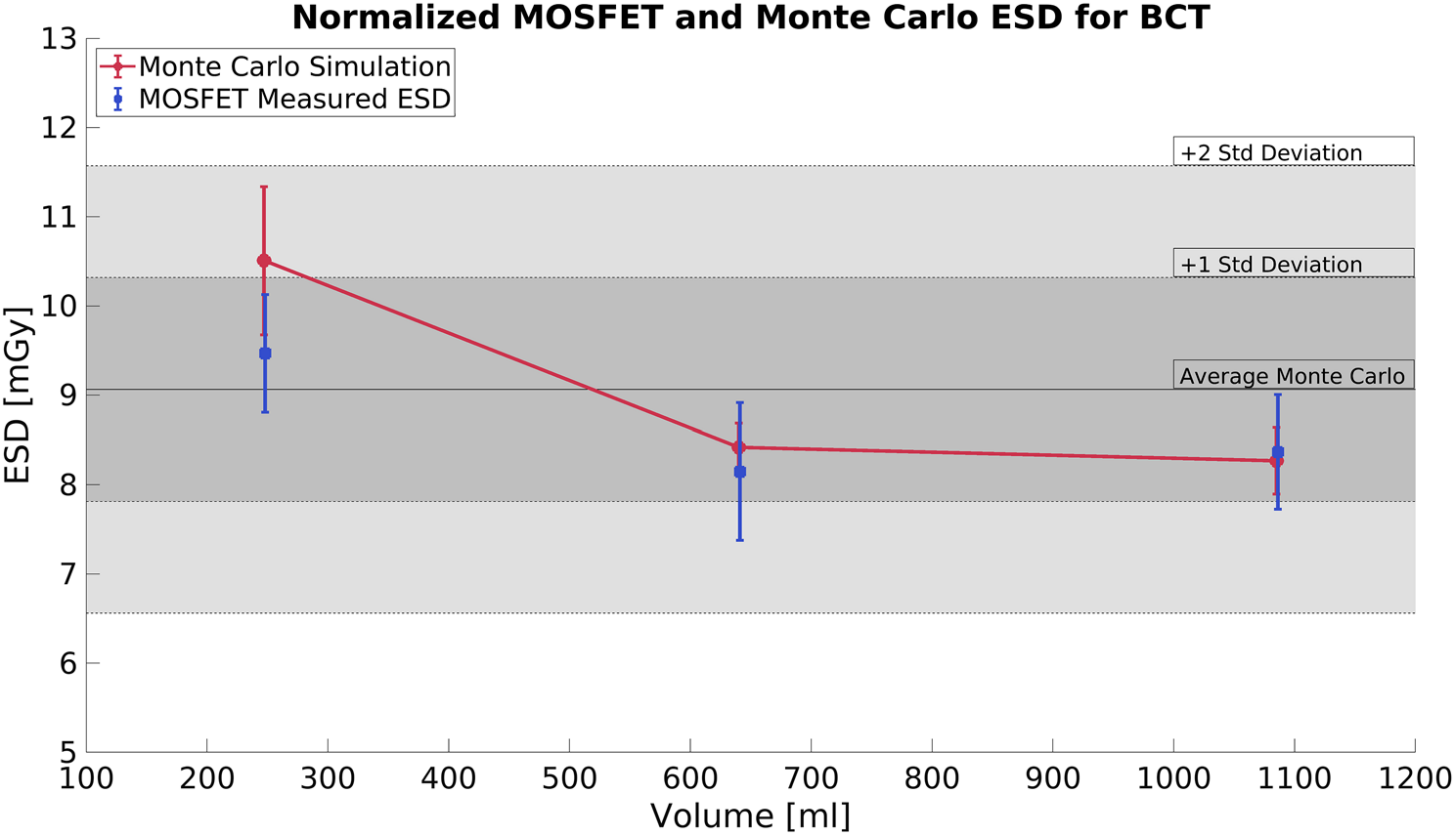
Validation of Entrance surface dose (ESD) for the breast CT data. To allow for comparison between different acquisition settings, all data points have been normalized to 25 mAs, and data for the different density mixtures have been averaged (due to the constant tube current used across different densities)

Figure 6 shows the ESD values for the BCT device, both measured and simulated. In the case of the BCT device, the tube output is not automatically adapted to the size and density of the specific breast, and therefore the ESD is, in first approximation, directly proportional to the tube output. Accordingly, given that the tube current was constant for all densities and only varied with size, the data points for the different density mixtures have been averaged. In the case of the large phantom (in which a higher tube current was used), in order to allow for direct comparison to the other phantoms, the measurement values were first normalised to 25 mA by linear scaling. Following normalisation, the data shows good agreement between simulated and measured values for all breast volumes, and all values lie within two standard deviations of the average ESD.

## Discussion

We have demonstrated that variable-shape, variable-density, fillable phantoms can be 3D-printed for use in both mammography and BCT devices. Specifically, uncompressed phantoms can be generated directly from exported BCT scans, converted to surface models in post-processing, and 3D-printed. Corresponding volume-matched compressed phantoms can be generated using the VICTRE pipeline, with post-processing to add a backing ‘chest wall’, prior to 3D printing.

Furthermore, we show that different phantom densities can be generated by varying filling mixture quantities. In the case of mammography, densities matching BI-RADS categories A - D could be measured, which included solid, semi-solid and liquid mixtures. The liquid mixture could be measured without risk to the device due to the enclosed morphology of the compressed breast phantoms (only a small filling opening at the top of the phantom), and due to the nature of the device (flat, exposed surface that could be easily cleaned in case of spillage). By contrast, measurement of the liquid state phantom was not possible on the BCT device due to a high risk of damage to the device in case of spillage, given the large opening and the components of the rotating gantry located directly below the phantoms. Additional safety measures were therefore employed, as previously discussed in [39].

Measurement results show that ESD increases with increasing glandularity and increases with increasing breast size, when an AEC system is available and activated. These results confirm the positive correlation between compressed breast thickness and dose reported in literature [16, 43–45], and are consistent with established breast dosimetry theory, in which the absorbed dose in glandular tissue is known to increase with compressed breast thickness and glandular fraction [46]. In particular, Dance et al. demonstrated that mean glandular dose (MGD) scales with entrance surface dose through conversion factors that depend on breast composition, thickness, and beam quality, providing a theoretical basis for the observed dependence of ESD on these parameters [46].

In the case of the mammography device used in this work, dose increases are primarily due to the intervention of the AEC system, meaning that the device settings (mAs, kVp) are automatically adjusted as a function of breast density and thickness. By contrast, in the BCT device the tube current is manually selected, resulting in the ESD depending only on the selected tube current for all phantoms, irrespective of density. These results are in line with results found on the same BCT device in previous work by our group using variable size measurement phantoms [39]. Although the MGD was not calculated in this work, an additional consideration is the calculation of MGD using homogenous versus anthropomorphic phantoms. Previous studies have shown that using homogeneous phantoms, MGD is overestimated compared to MGD in anthropomorphic phantoms, and that the level of over-estimation varies between different modalities, with overestimation being higher for mammography devices [47–50].

Overall, the phantom measurements display the expected properties, namely dependency on size and density for the mammography unit (which has AEC), and proportionality to tube current alone for BCT. Additionally, measurements also exhibited a good match with the Monte Carlo simulations performed with a software-defined density. The agreement between simulation and the purpose built phantoms across densities demonstrates that our method allows for good control over the phantom properties.

The ability to directly compare ESD between mammography and BCT demonstrates the usefulness of the 3D-printed phantoms; if completed over a larger range of sizes and densities, results such as those presented could be used to improve the referral guidelines for breast exams and minimise the overall dose exposure in cases where both BCT and mammography devices are available.

Although promising, there are several limitations of this work. First, the liquid nature of the BI-RADS category A phantom precluded measurement in the BCT device, and future work will therefore focus on improving the phantom shape to better contain liquid mixtures and prevent accidental spillage. In addition, in this work, the BCT device did not use AEC. In clinical use, scan length and tube current settings are manually adjusted according to manufacturer recommendations to optimise image quality. Therefore, the actual dose may vary if very different values are selected by the operators. Future work should therefore also include visual assessment of the phantoms on the scans, or repeat scans, when AEC becomes available for the BCT device. In addition, future phantoms may include additional inclusions in the filling mixture, or adding structures into the 3D printing files, in order to more realistically simulate the variability of human breast tissue.

## Conclusion

We have successfully demonstrated that both uncompressed and compressed breast phantoms of varying size can be generated, 3D-printed, and filled with variable density mixtures for comparison of radiation dose measurements across different breast imaging devices. The phantoms exhibit the expected properties in terms of X-ray attenuation, and direct ESD measurements were in agreement with Monte Carlo simulations, as well as in line with previous measurements performed with similar devices. The method described here therefore provides a promising technique for generating phantoms with well-controlled, reproducible properties that can be used for comparative evaluation of dose deposition of breast imaging devices, which could in turn impact the future development of Quality Assurance protocols and clinical recommendations for device settings.

## Electronic supplementary material

Below is the link to the electronic supplementary material.

Supplementary Material 1

## Data Availability

Data availability: The generated phantom files are available on GitHub at: https://github.com/USZ-MPER Code availability: Some scripts used in this work to generate and process the virtual phantoms are available on GitHub at: https://github.com/USZ-MPER.

## Code availability

Some scripts used in this work to generate and process the virtual phantoms are available on GitHub at: https://github.com/USZ-MPER.
