# Supplementary material for: Design and measurement of 3D-printed variable-morphology and variable-density breast phantoms for mammography and breast CT dose assessment
Source: 3D Print Med. 2026 Jul 11;12:23. doi: 10.1186/s41205-026-00335-9 (PMC13374221; doi:10.1186/s41205-026-00335-9)
Supplement: Supplementary file 1 — Supplementary Material 1 [file 41205_2026_335_MOESM1_ESM.pdf]

Appendix A Measurement Images

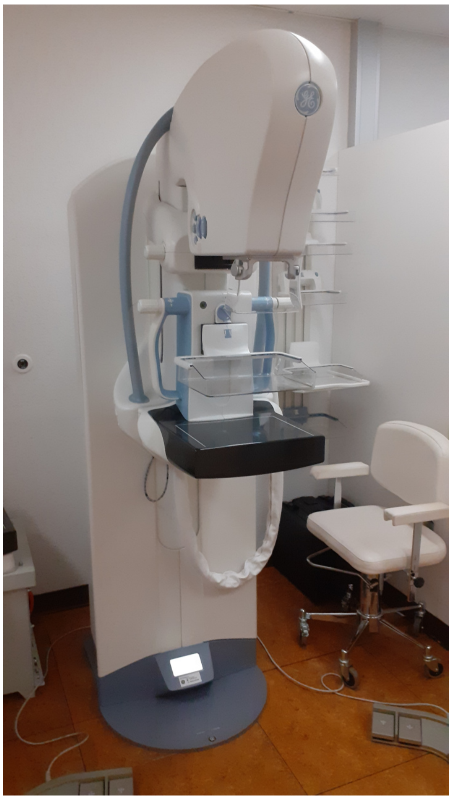

Fig. A1 The mammography unit used in this work (Senographe Essential, GE Healthcare, DE).

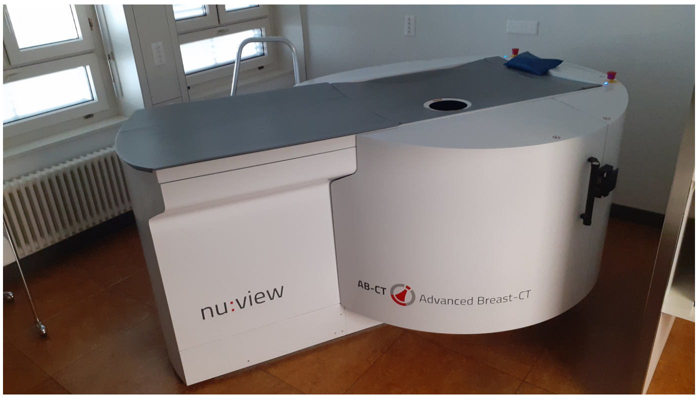

Fig. A2 The breast CT unit used in this work (nu:view, Advanced Breast CT).

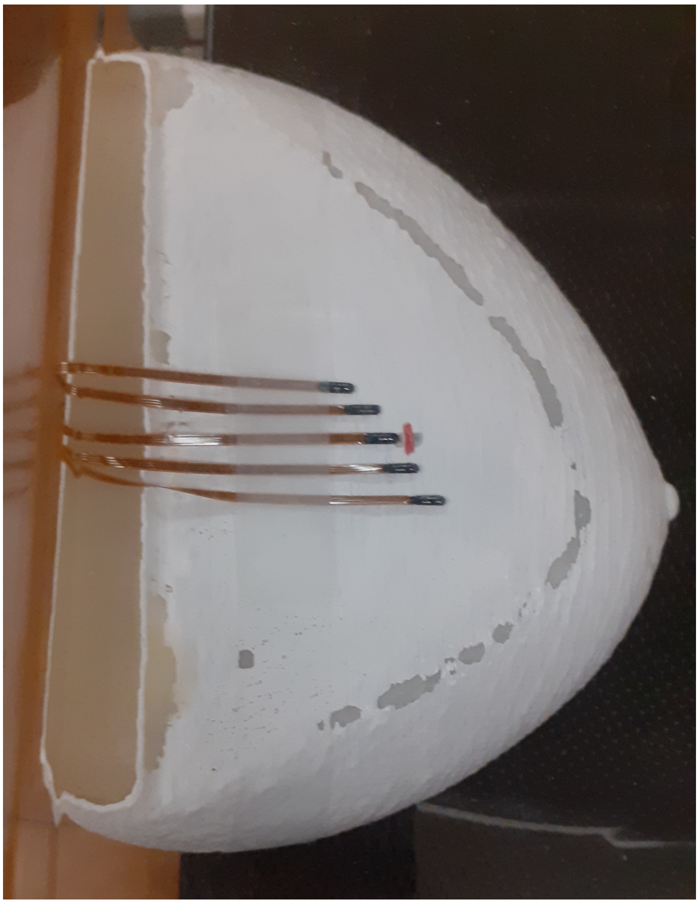

Fig. A3 Close up image of the filled compressed phantom with dosimeter placement.

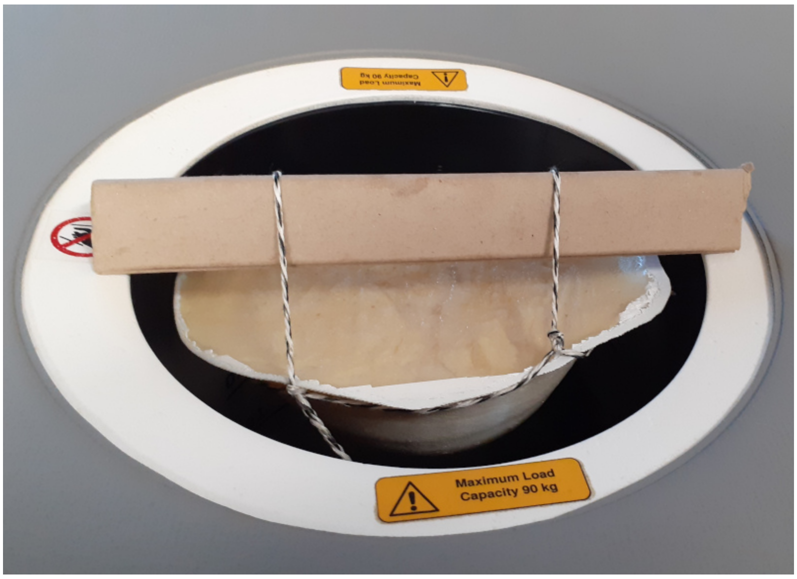

Fig. A4 Filled breast phantom suspended over BCT scanner.
